# CONTI-CrackNet: A Continuity-Aware State-Space Network for Crack Segmentation

**DOI:** 10.3390/s25226865

**Published:** 2025-11-10

**Authors:** Wenjie Song, Min Zhao, Xunqian Xu

**Affiliations:** 1 School of Information Science and Technology, Nantong University, Nantong 226019, China; wenjiesong@stmail.ntu.edu.cn; 2 School of Artificial Intelligence and Computer Science, Nantong University, Nantong 226019, China; 3 School of Transportation and Civil Engineering, Nantong University, Nantong 226019, China; xunqian_xu@ntu.edu.cn

**Keywords:** crack segmentation, segmentation, Mamba, deep learning, lightweight networks, feature extraction

## Abstract

Crack segmentation in cluttered scenes with slender and irregular patterns remains difficult, and practical systems must balance accuracy and efficiency. We present CONTI-CrackNet, which is a lightweight visual state-space network that integrates a Multi-Directional Selective Scanning Strategy (MD3S). MD3S performs bidirectional scanning along the horizontal, vertical, and diagonal directions, and it fuses the complementary paths with a Bidirectional Gated Fusion (BiGF) module to strengthen global continuity. To preserve fine details while completing global texture, we propose a Dual-Branch Pixel-Level Global–Local Fusion (DBPGL) module that incorporates a Pixel-Adaptive Pooling (PAP) mechanism to dynamically weight max-pooled responses and average-pooled responses. Evaluated on two public benchmarks, the proposed method achieves an F1 score (F1) of 0.8332 and a mean Intersection over Union (mIoU) of 0.8436 on the TUT dataset, and it achieves an mIoU of 0.7760 on the CRACK500 dataset, surpassing competitive Convolutional Neural Network (CNN), Transformer, and Mamba baselines. With 512 × 512 input, the model requires 24.22 G floating point operations (GFLOPs), 6.01 M parameters (Params), and operates at 42 frames per second (FPS) on an RTX 3090 GPU, delivering a favorable accuracy–efficiency balance. These results show that CONTI-CrackNet improves continuity and edge recovery for thin cracks while keeping computational cost low, and it is lightweight in terms of parameter count and computational cost.

## 1. Introduction

Cracks are among the most common and harmful defects [1]. They weaken the load-bearing capacity and durability of structures, increase maintenance costs, and pose public-safety risks. Cracks manifest in diverse materials and structures, such as pavements, bricks, and tiles [2]. Owing to irregular shapes, large-scale variations, and complex textures, accurate crack recognition remains challenging. Traditional crack detection still relies on manual inspection [3]. This process is slow, costly, and subjective, which often leads to missed cases and inconsistent results and cannot meet large-scale application needs. Early segmentation methods mainly used digital image processing, such as edge detection, clustering, thresholding, and morphological operations [4,5,6,7], but their performance is limited in the presence of noise.

To overcome these limitations, researchers introduced Convolutional Neural Networks (CNNs) for crack segmentation [8]. Although prior studies expanded the effective receptive field through pyramid pooling [9], employed deformable convolutions to adaptively focus on relevant regions [10], and incorporated lightweight attention in SegNeXt [11] to highlight local features while reducing computational overhead, CNNs remain constrained by local convolutions [12], which limits the modeling of long-range dependencies and complex semantic relations. In view of these shortcomings, Transformer-based methods have been applied to segmentation tasks, including hierarchical encoders coupled with Multi-layer Perceptron (MLP) decoders for cross-level feature aggregation [13] and CNN–Transformer dual-path architectures that exploit complementary strengths [14]. However, Transformers typically involve large parameter scales and substantial computational cost [15], which restricts deployment on resource-constrained devices. Moreover, MaskSup [16] performs context modeling for image segmentation via random masking, thereby improving segmentation performance without incurring any additional inference overhead.

To retain both global and local modeling while controlling computational cost, researchers have begun exploring state-space models for vision tasks [17]. Mamba is an efficient model in this class, employing selective state spaces to model long sequences in linear time [18] and thereby delivering strong global-context capture with high computational efficiency. Building on this idea, many studies have extended Mamba to computer vision. For example, VMamba [19] divides an image into a sequence of patches and employs Visual State-Space (VSS) blocks for multi-directional scanning to better capture complex inter-patch dependencies. PlainMamba [20] removes the hierarchical structure and adopts continuous 2D scanning, simplifying the network and improving inference efficiency. MambaIR [21] incorporates local enhancement and channel attention and demonstrates improvements in image restoration. CSMamba [22] combines a CNN encoder with a Mamba decoder for remote-sensing image segmentation.

Overall, these studies indicate that Mamba offers clear advantages for global-information modeling. However, despite progress in image restoration and remote sensing [23,24,25], in-depth investigations of crack segmentation remain limited. A few studies have introduced Mamba into crack segmentation. For example, CrackMamba [26] adopts a Mamba-based encoder and a Snake-scan mechanism to strengthen global modeling; MambaCrackNet [27] incorporates novel visual Mamba blocks, effectively capturing global semantic relations while reducing computational cost. Cracks exhibit a slender, irregular morphology with fuzzy boundaries, which requires models to preserve spatial continuity while balancing pixel-level local details and global texture. At present, crack segmentation primarily relies on architectures based on CNNs or Transformers [28,29,30]. The former (e.g., SFIAN [31]) excels at detailed feature extraction, whereas the latter (e.g., CT-crackseg [32]) achieves gains in boundary modeling. Yet CNNs are limited by the locality of convolutions, whereas Transformers incur high computational cost due to attention [33]. Consequently, achieving a favorable balance between segmentation quality and computational efficiency remains an open problem.

To address the above challenges, we propose CONTI-CrackNet. Our main contributions are outlined below:

1. CONTI-CrackNet architecture: a cascaded and lightweight crack segmentation network that effectively represents complex crack morphology while markedly reducing computation.
2. Multi-Directional Selective Scanning Strategy (MD3S): efficient long-range modeling that characterizes crack continuity and shape from multiple directions combined with the Bidirectional Gated Fusion (BiGF) module to alleviate directional bias.
3. Dual-Branch Pixel-Level Global–Local Fusion (DBPGL) module: a Pixel-Adaptive Pooling (PAP) mechanism that balances max-pooled and average-pooled features at each pixel, preserving edge fidelity while improving global connectivity.

## 2. Methods

### 2.1. Network Architecture

**Figure 1:**
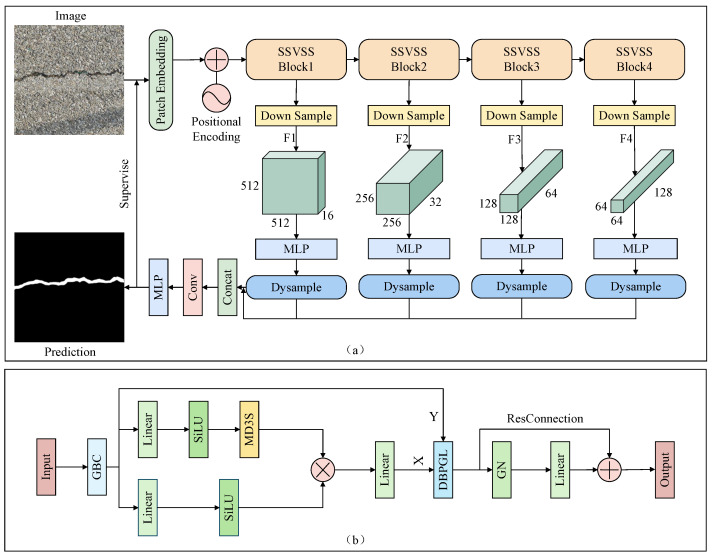
Overall framework of CONTI-CrackNet: (**a**) CONTI-CrackNet, (**b**) SSVSS block.

This paper proposes a new crack-segmentation model, CONTI-CrackNet; its overall structure is shown in Figure 1a. The model addresses multiple crack properties: it enhances shape modeling, captures global information about crack continuity, and preserves the local details of fine cracks. Building on this design, the model employs a Multi-Directional Selective Scanning Strategy (MD3S), a Dual-Branch Pixel-Level Global–Local Fusion (DBPGL) module, and a Pixel-Adaptive Pooling (PAP) mechanism to achieve fine-grained segmentation in complex scenes.

The input is an RGB image of shape (3, H, W), where 3 denotes the channel count and H and W denote the image height and width, respectively. We first partition the image into $N=\frac{H}{h}\cdot \frac{W}{w}$ patches, where h and w are the height and width of each patch, and add positional encodings to each patch to preserve spatial information. The patch sequence is then fed into an Structure-Strengthened Visual State-Space (SSVSS) block for feature modeling. As shown in Figure 1b, the SSVSS block first applies a Gated Bottleneck Convolution (GBC) [34] module to extract detailed features and then splits the stream into two branches. The lower branch expands the channels via a linear layer and applies SiLU. The upper branch applies a linear transform and activation, and then it introduces an MD3S to better capture the crack shape and structure. The outputs of the two branches are fused by element-wise multiplication and projected back to the original channel size via a linear layer.

To improve both local detail and global semantics, we further design DBPGL. The MD3S-processed features and the corresponding original features are fed into this module. This module employs PAP to fuse max-pooling and average-pooling features at the pixel level, thereby enhancing both global and local representations. Residual connections are added in the SSVSS block to preserve original details during fusion and mitigate gradient vanishing. During feature extraction, the network produces four feature maps at different scales: low-level maps primarily encode spatial details, whereas high-level maps carry richer semantic information. These features gradually integrate spatial structure and contextual information. Afterward, an MLP aligns the four scales to a common channel width, and dynamic upsampling [35] restores them to the original image resolution. Finally, all feature maps are concatenated and further processed by a convolutional layer and an MLP to yield the final crack-segmentation map.

### 2.2. Multi-Directional Selective Scanning Strategy (MD3S)

**Figure 2:**
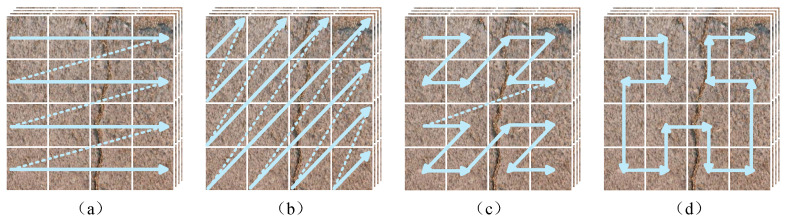
Four common single-strategy scans: (**a**) parallel scans, (**b**) diagonal scans, (**c**) Z-order scans, (**d**) Hilbert scans.

In recent years, two-dimensional scanning strategies have demonstrated strong feature-modeling capability in image analysis tasks. However, common scanning modes in visual Mamba models include parallel, diagonal, Z-order, and Hilbert scans [36,37]. Each mode follows a single strategy (Figure 2). Parallel scans better capture long-range dependencies along the horizontal and vertical directions; diagonal and Z-order scans are more suitable for modeling global information along diagonal paths; and Hilbert scans preserve spatial continuity to some extent but are weaker in capturing global information. Thus, single-strategy modes are limited when handling complex structures and cannot fully represent slender, irregular cracks across multiple directions [38].

**Figure 3:**
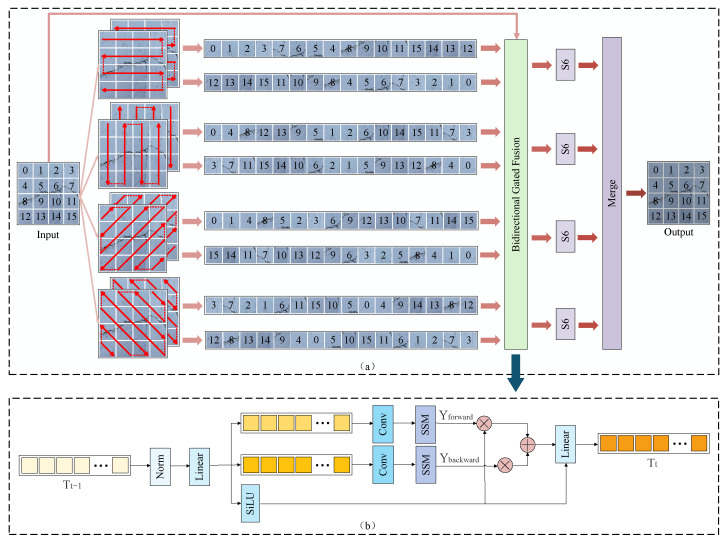
Architecture of MD3S: (**a**) MD3S, (**b**) BiGF.

To address this limitation, we propose the Multi-Directional Selective Scanning Strategy (MD3S). MD3S scans along four directions: horizontal, vertical, main-diagonal, and anti-diagonal. It performs forward and backward passes in each direction to obtain four bidirectional feature sequences (Figure 3a). To reduce redundancy and emphasize complementary information between the bidirectional sequences [39], we introduce a Bidirectional Gated Fusion (BiGF) module (Figure 3b), which adaptively assigns weights to the forward and backward paths and fuses them:

(1) $$\sigma =\mathrm{SiLU}\left(\mathrm{Linear}\left(\mathrm{Norm}\left(T_{t-1}\right)\right)\right)$$

(2) $$T_{t}=\mathrm{Linear}\left(\sigma \cdot Y_{\mathrm{forward}}+\left(1-\sigma \right)\cdot Y_{\mathrm{backward}}\right)+T_{t-1}$$

where $T_{t-1}$ is the original feature and $T_{t}$ is the output feature; Norm denotes normalization, Linear denotes a linear projection, and SiLU is the activation function; $Y_{\mathrm{forward}}$ and $Y_{\mathrm{backward}}$ denote the features from the forward and backward paths, respectively.

After the gated fusion, the four fused sequences are passed to the S6 block [18]. A subsequent scan–merge operation concatenates the sequences from different directions and maps them back to the original spatial resolution, yielding a representation that preserves multi-directional structural awareness and global-context modeling.

### 2.3. Dual-Branch Pixel-Level Global–Local Fusion (DBPGL) Module

To preserve fine-grained details from the original sequence while fusing the crack texture patterns produced by MD3S, we propose the DBPGL module. DBPGL incorporates a Pixel-Adaptive Pooling (PAP) mechanism within a dual-branch design. It performs dynamic weighting at the pixel level, thereby strengthening the SSVSS module’s capacity to model and learn crack features.

**Figure 4:**
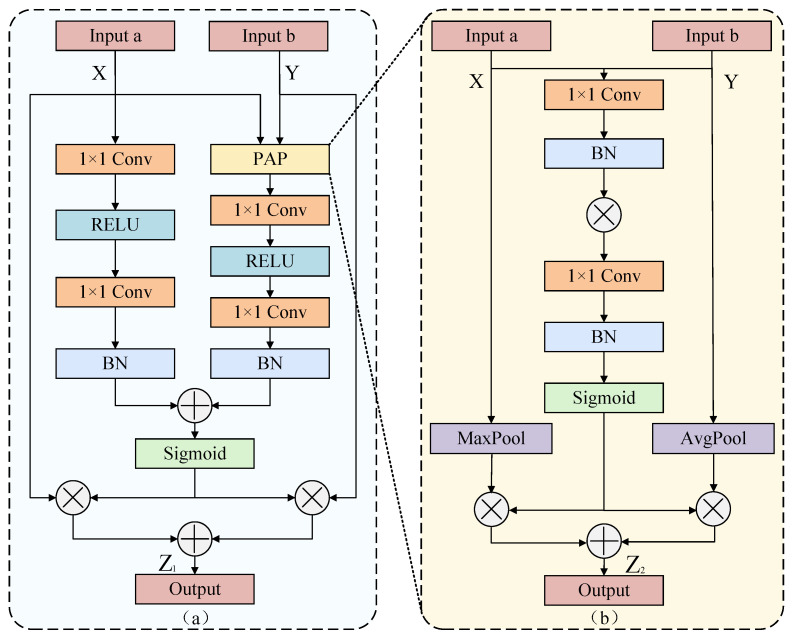
Structures of DBPGL and PAP: (**a**) DBPGL; (**b**) PAP.

As shown in Figure 4a, DBPGL fuses two inputs using a dual-branch structure, focusing on local detail preservation and global texture completion. In the left branch, the input feature passes through a 1 × 1 convolution for channel reduction, which is followed by ReLU to introduce nonlinearity and enhance representation. A 1 × 1 convolution then restores the original channel number. Batch normalization is applied to improve training stability and generalization. The right branch employs PAP to complement global structural information related to crack continuity. Finally, the outputs of the two branches are fused. Element-wise operations compute per channel attention weights via a sigmoid function, which enables adaptive importance assignment to the two branches. The fusion can be written as the equations below.

(3) $$X_{l}=\mathrm{BN}\left(\mathrm{Conv}\left(\mathrm{ReLU}\left(\mathrm{Conv}\left(X\right)\right)\right)\right)$$

(4) $$Y_{g}=\mathrm{BN}\left(\mathrm{Conv}\left(\mathrm{ReLU}\left(\mathrm{Conv}\left(Y\right)\right)\right)\right)$$

(5) $$Z_{1}=\mathrm{Sigmoid}\left(X_{l}+Y_{g}\right)\cdot X+\left(1-\mathrm{Sigmoid}\left(X_{l}+Y_{g}\right)\right)\cdot Y$$

where X and Y denote input a and input b; Conv denotes the 1 × 1 convolution; ReLU denotes the activation function; BN denotes batch normalization; Sigmoid denotes the activation function. $X_{l}$ denotes the output of the left branch of the module, $Y_{g}$ represents the output of the right branch, and $Z_{1}$ is the fused output obtained by weighting and combining the features from both branches.

### 2.4. Pixel-Adaptive Pooling (PAP) Mechanism

In feature extraction, max pooling and average pooling are often used together because they are complementary. Max pooling focuses on high-response regions, whereas average pooling integrates global background information and yields a balanced representation [40]. To leverage both, we design a pixel-level adaptive fusion strategy within PAP, as illustrated in Figure 4b. Specifically, the input features X and Y are split into two paths and processed by max pooling and average pooling to obtain complementary spatial features. During two-branch feature fusion, a fixed rule cannot adapt to crack structures at the pixel scale. We introduce the Pixel Attention Mechanism (PAM) [41] to enable adaptive, pixel-level fusion. It assigns weights to the two complementary branches at each spatial location, preserving fine details and maintaining global connectivity. This mechanism applies a 1 × 1 convolution followed by batch normalization to each path, performs element-wise multiplication for interaction, and then applies another 1 × 1 convolution and batch normalization. The output is passed through a sigmoid to produce a gating coefficient σ, which encodes the dynamic preference between the two branches. Finally, the pixel attention weights are applied to the two features. Fusion is completed by weighted multiplication and element-wise addition, which is formulated as shown below:

(6) $$\sigma =\mathrm{Sigmoid}\left(\mathrm{BNConv}\left(\mathrm{BNConv}\left(V_{a}\right)\cdot \mathrm{BNConv}\left(V_{b}\right)\right)\right)$$

(7) $$Z_{2}=\sigma \cdot X_{\max}+\left(1-\sigma \right)\cdot Y_{\mathrm{avg}}$$

where $V_{a}$ and $V_{b}$ are pixels from the two-branch feature maps; BNConv denotes a 1×1 convolution followed by batch normalization, i.e., BNConv()=BN(Conv()); Sigmoid is the activation function; $X_{\max}$ and $Y_{\mathrm{avg}}$ are the feature maps produced by max pooling and average pooling, respectively; and $Z_{2}$ is the fused output obtained by weighting and combining the features from both branches. With this design, the network fuses important local features with global context and can adapt, at the pixel level, the contribution of different spatial locations, which improves the modeling of complex crack structures.

## 3. Results

### 3.1. Datasets

**Figure 5:**
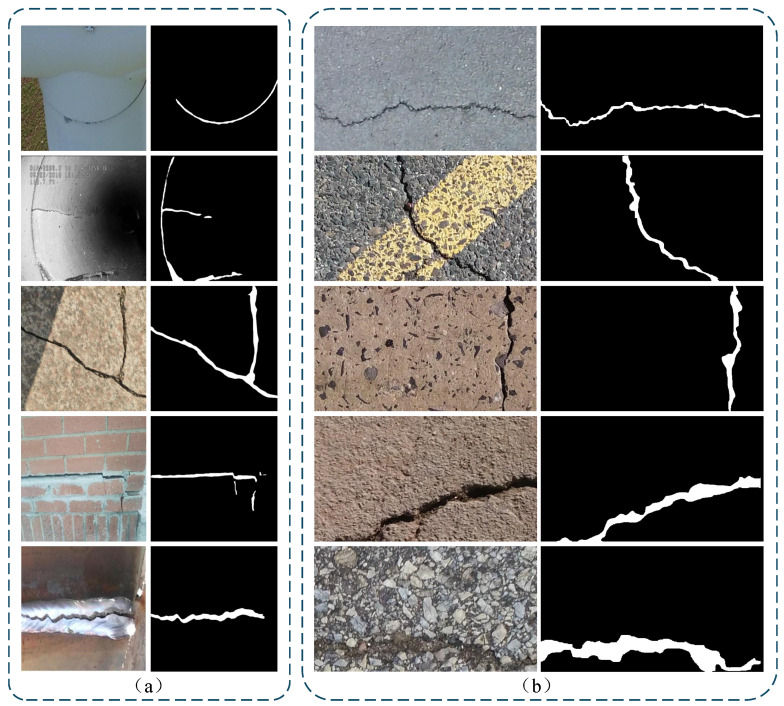
Sample images and ground-truth masks from the datasets used in this study: (**a**) TUT, (**b**) CRACK500.

We validate our approach on two public benchmark datasets for crack segmentation: TUT [42] and CRACK500 [43]. TUT covers diverse scenes with complex backgrounds, which helps assess the model’s robustness and generalization across scenes and under strong noise. CRACK500 is a standard pavement crack benchmark that enables fair comparison with prior methods. Table 1 reports the main characteristics and split schemes of the datasets, while Figure 5 presents representative images and the corresponding ground truth masks.

1. TUT [42]: Unlike datasets with simple backgrounds, TUT contains dense and cluttered scenes with diverse crack shapes. It includes 1408 RGB images from eight representative scenes: bitumen, cement, bricks, runways, tiles, metal, generator blades, and underground pipelines. Of these, 1270 images were collected in-house using mobile phones, and 138 images were sourced from the internet.
2. CRACK500 [43]: Proposed by Yang et al., the original dataset contains 500 bitumen crack images at 2000 × 1500 resolution, which were all captured by mobile phones. Because the dataset is small, images are cropped into 16 nonoverlapping patches, and only samples with more than 1000 crack pixels are retained. After this processing, each patch has a resolution of 640 × 320. Data augmentation increases the total to 3368 images, and each sample is paired with a per-pixel binary mask.

### 3.2. Evaluation Metrics

To comprehensively evaluate the proposed segmentation model, we use several common metrics: Optimal Dataset Scale (ODS), Optimal Image Scale (OIS), Precision (P), Recall (R), F1 score (F1), and mean Intersection over Union (mIoU). The ODS evaluates the model on the whole dataset under a single fixed threshold m. The OIS evaluates performance when an optimal threshold n is chosen for each image. The F1, as the harmonic mean of P and R, balances both and is widely used to assess overall robustness and reliability in crack segmentation. In addition, mIoU quantifies spatial accuracy by the overlap between the predicted mask and the ground-truth annotation. They are defined as shown below:

(8) $$\mathrm{ODS}=\max_{m}\frac{2\cdot P_{m}\cdot R_{m}}{P_{m}+R_{m}}$$

(9) $$\mathrm{OIS}=\frac{1}{N}\sum_{i=1}^{N}\max_{n}\frac{2\cdot P_{n,i}\cdot R_{n,i}}{P_{n,i}+R_{n,i}}$$

(10) $$F_{1}=\frac{2\cdot P\cdot R}{P+R}$$

(11) $$\mathrm{mIoU}=\frac{1}{N+1}\sum_{i=0}^{N}\frac{p_{ii}}{\sum_{j=0}^{N}p_{ij}+\sum_{j=0}^{N}p_{ji}-p_{ii}}$$

where N is the number of classes; i indexes the ground-truth class and j denotes the predicted class. Let $p_{ij}$ denote the number of pixels of ground-truth class i predicted as class j (thus, $p_{ii}$ are the true positives for class i). In this case, N=1.

### 3.3. Implementation Details and Training

The proposed network is implemented in PyTorch v1.13.1 and trained on an Intel Xeon Platinum 8336C CPU. AdamW is used with an initial learning rate of 5 × 10^−4^ and the PolyLR scheduler for dynamic adjustment. The weight decay is 0.01, and the random seed is 42. Training runs for 50 epochs. All input images are resized to 512 × 512 pixels, and the batch size is 8. After each epoch, performance on the validation set is measured, and the checkpoint with the best validation score is kept for later testing.

A joint loss that combines Binary Cross-Entropy (BCE) and Dice loss [44] is adopted. BCE measures per-pixel classification accuracy, while Dice focuses on the overlap between the predicted mask and the ground truth, improving coherence and completeness. The combined loss is shown below:

(12) $$L=\alpha \;\cdot L_{\mathrm{BCE}}+\left(1-\alpha \right)\;\cdot L_{\mathrm{Dice}}$$

where $L_{\mathrm{BCE}}$ is the BEC, $L_{\mathrm{Dice}}$ is the Dice loss, and α controls the weights of the two terms. As shown in Table 2, we conduct a sensitivity study of the loss-weight hyperparameter α on the TUT dataset; the results indicate that α = 0.2 achieves the best performance.

### 3.4. Experimental Results

We evaluate the proposed model against seven representative baseline networks: SFIAN [31], SegNeXt [11], Crackmer [14], SegFormer [13], CT-crackseg [32], CSMamba [22], and PlainMamba [20]. On two public datasets, we make a systematic comparison between these methods and our model. Specifically, SFIAN and SegNeXt are built on CNNs; Crackmer, SegFormer, and CT-crackseg adopt the Transformer architecture; CSMamba and PlainMamba follow the Mamba framework.

#### 3.4.1. Experimental Results on Dataset TUT

On the TUT dataset, CONTI-CrackNet achieves the best results across all metrics. As shown in Table 3, our model reaches an F1 of 0.8332 and an mIoU of 0.8436, outperforming architectures based on CNNs, Transformers, and Mamba. Compared with SegNeXt, F1 improves by 0.0815, indicating a stronger modeling of thin and low-contrast cracks, with fewer breaks and missed segments along long, slender structures. Compared with the best Transformer baseline, CT-crackseg, R increases by 0.0248, confirming advantages in accuracy. Compared with strong Mamba-based baselines, the proposed method attains higher accuracy; relative to PlainMamba, mIoU increases by 0.0120, demonstrating clear competitiveness within the same architectural class. In addition, a standard deviation analysis was conducted on the TUT dataset. The standard deviations of ODS, OIS, F1, P, R, and mIoU are 0.0114, 0.0106, 0.0065, 0.0043, 0.0102, and 0.0071, respectively. These results demonstrate that the proposed model performs well and exhibits strong engineering reliability.

**Figure 6:**
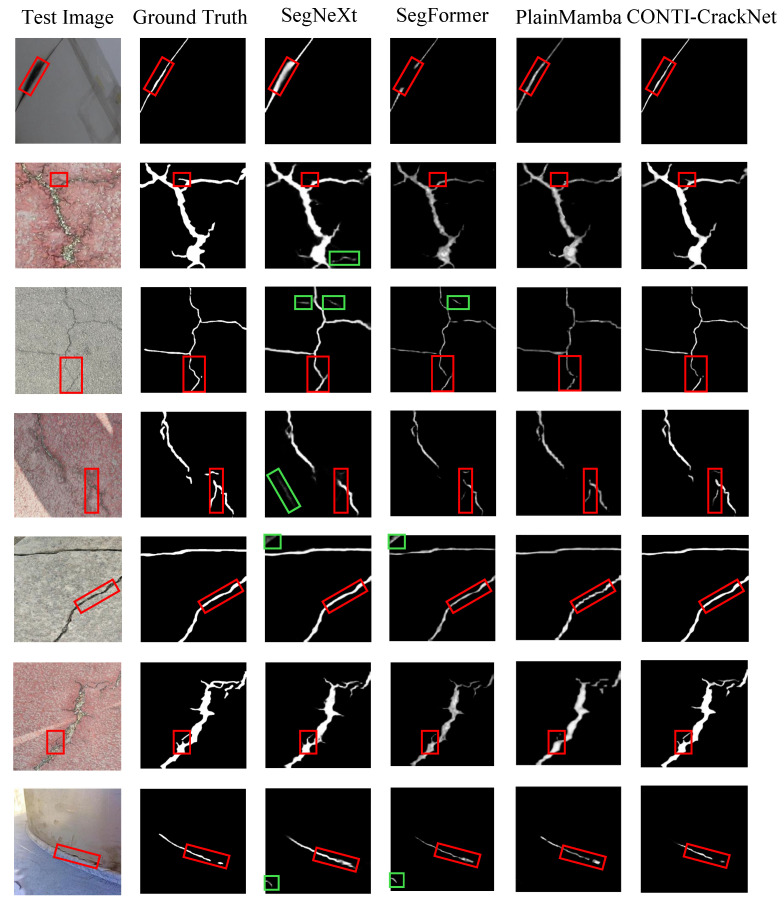
Typical visual comparison on the TUT dataset using four methods. Red boxes highlight key details; green boxes mark wrongly identified regions.

Beyond the numbers, our visual results also show clear gains. As shown in Figure 6, in scenes with complex backgrounds and uneven lighting, SegNeXt and SegFormer tend to produce false cracks and false positives, while CONTI-CrackNet suppresses background noise. For very thin cracks or low contrast, CNN and Transformer methods often show broken or missed segments, but our method restores complete crack shapes. For long and curved cracks, PlainMamba still shows blurred edges and local breaks, while CONTI-CrackNet keeps global continuity and sharp boundaries. These improvements come from the MD3S, which models crack continuity, and the DBPGL module, which fuses global and local features. Together, they enable robust segmentation in complex scenes and demonstrate the effectiveness and practical value of our method.

#### 3.4.2. Experimental Results on Dataset CRACK500

On the CRACK500 dataset, the results further verify the effectiveness of our method. As shown in Table 4, CONTI-CrackNet attains an mIoU of 0.7760, outperforming all compared methods on every metric. Compared with the second-best PlainMamba, our model improves by 0.0181 on mIoU, respectively. Against the CNN and Transformer baselines, the gains are larger, especially on F1 and mIoU, which shows stronger robustness in preserving overall crack structure and regional consistency. On CRACK500, the standard deviations for ODS, OIS, F1, P, R, and mIoU are 0.0057, 0.0102, 0.0091, 0.0146, 0.0052, and 0.0119, respectively. This finding demonstrates the consistency and reliability of the proposed model across diverse evaluation conditions.

**Figure 7:**
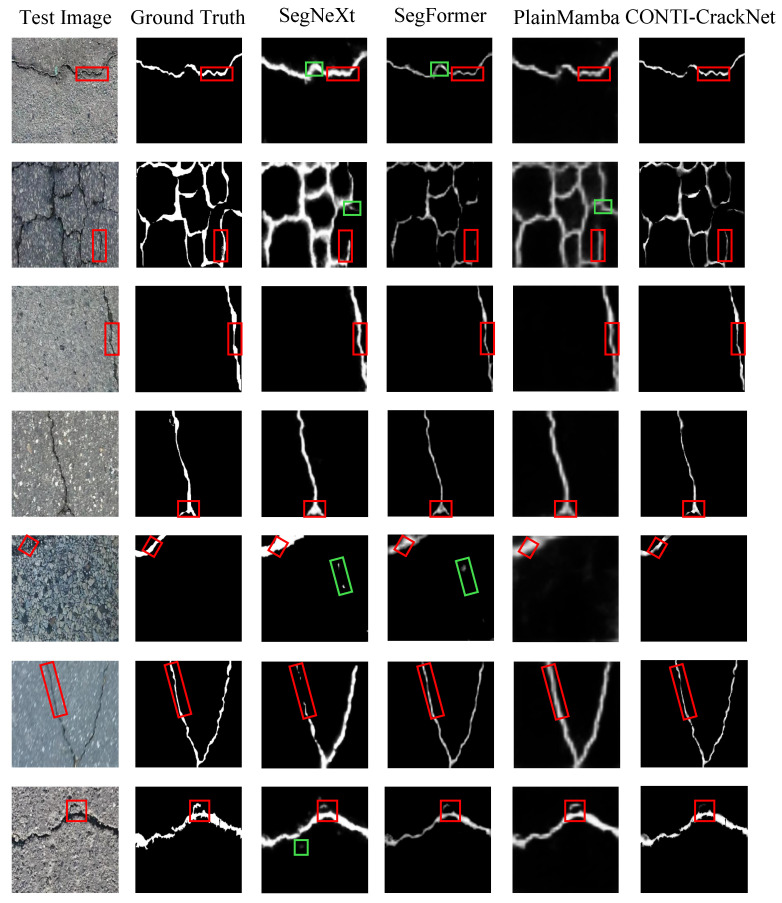
Typical visual comparison on the CRACK500 dataset using four methods. The red boxes highlight key details; the green boxes mark wrongly identified regions.

The visual results in Figure 7 also show clear advantages. In scenes with complex backgrounds and strong texture noise, CNN and Transformer models such as SegNeXt and SegFormer often produce false positives or fake cracks, as seen in the first and fifth rows of Figure 7. When crack shapes are complex, Mamba methods can still show over segmentation or blurry boundaries, as illustrated in the second row. In contrast, CONTI-CrackNet accurately extracts the main crack skeleton in most cases and maintains sharp edges and global continuity; even under high noise or low contrast, it avoids several false segmentations. Although CONTI-CrackNet does not perfectly recover every tiny crack, its results are closer to the ground truth and contain much less noise overall.

### 3.5. Ablation Study

To verify the effectiveness of the proposed modules, we conduct a systematic ablation study on the TUT dataset. Specifically, we replace, enable, or disable the proposed modules to systematically assess each module’s impact on segmentation performance. This design quantifies the contributions of each component and the proposed scanning strategy and further confirms the soundness and effectiveness of the approach.

#### 3.5.1. Ablation Study of Components

We further conduct ablation studies on the modules introduced in SSVSS. As summarized in Table 5, the full configuration integrating MD3S, DBPGL, and PAP attains the best overall metrics, validating its effectiveness for crack segmentation. The baseline excludes all proposed modules and only employs the standard SS2D scanning [17] with element-wise addition (ElemAdd) for fusion. On this basis, introducing MD3S increases the mIoU to 0.8350, indicating that MD3S effectively captures directional continuity and multi-orientation morphology.

In the comparison of fusion strategies, both ElemAdd and SKNet [45] underperform DBPGL. With the addition of PAP, the metrics further improve to 0.8436, suggesting that the combination of DBPGL and PAP enables fine-grained, pixel-adaptive gating between the two branches. Moreover, the global–local dual-branch design better suits crack scenarios, where max-pooled and average-pooled features are complementarily fused at the pixel level, thereby preserving the connectivity of slender and discontinuous cracks while suppressing texture noise.

In summary, MD3S, DBPGL, and PAP exhibit clear divisions of labor and synergy: MD3S provides direction-aware global modeling and continuity completion; DBPGL together with PAP delivers complementary global–local fusion with pixel-level adaptive weighting to refine fusion decisions. Their joint effect leads to substantial performance gains in crack segmentation.

#### 3.5.2. Ablation on Scanning Strategies

As shown in Table 6, we compare four directional scanning strategies under the same settings. In this experiment, we disable both DBPGL and PAP, and we perform feature fusion using only ElemAdd. Using parallel serpentine scanning (ParaSpn), with forward and backward traversals along the horizontal and vertical directions, the model achieves F1 = 0.8063. Using diagonal serpentine scanning (DiagSpn) [37], with forward and backward traversals along the main and anti-diagonal directions, the model attains F1 = 0.8077, which remains relatively low. Combining the two (ParaSpn + DiagSpn), employing both parallel and diagonal serpentine scans, raises the F1 to 0.8127 and the mIoU to 0.8260, indicating that multi-directional cues benefit crack segmentation. Furthermore, after introducing the proposed MD3S, the model achieves new best results across all metrics. Compared with the combination of ParaSpn and DiagSpn, F1 and P improve by 0.0141 and 0.0252, respectively. These results indicate that the proposed strategy captures multi-direction features and strengthens the modeling of complex crack structures. Compared with a single scanning strategy, our method builds forward–backward (bidirectional) sequences in four directions: horizontal, vertical, main diagonal, and anti-diagonal. It then performs direction-wise fusion. This lets the network learn pixel-level adaptive preferences. Compared with simple multi-direction stacking, the per-direction bidirectional fusion better fits the modeling needs of multi-scale and multi-directional geometric features. It yields superior performance on crack.

#### 3.5.3. Ablation Study of the Attention Mechanism in the PAP Module

To substantiate the necessity of the proposed Pixel Attention Mechanism (PAM), we conducted a controlled comparison under identical experimental settings (Table 7). In this study, MD3S and DBPGL were kept enabled, and only the weight-generation unit in the fusion stage was swapped between the Convolutional Block Attention Module (CBAM) [46] and PAM. The results show that the model with PAM delivers the best performance, indicating that pixel-level, adaptive gating weights better discriminate thin cracks from background textures than CBAM, effectively reducing false positives and breakages and achieving finer pixel-wise segmentation.

### 3.6. Complexity Analysis

Table 8 reports the comparison under a fixed input size of 512 × 512. Our method requires 24.22 G floating point operations (GFLOPs), has 6.01M parameters (Params), and runs at 42 frames per second (FPS). Compared with the lightweight Crackmer [14], our model is slightly heavier but delivers a clear accuracy gain, achieving a better accuracy–efficiency trade-off. At the same time, compared with larger and more complex networks such as CSMamba [22] and SegFormer [13], our method uses fewer GFLOPs and Params and offers much faster inference. In sum, CONTI-CrackNet provides high segmentation accuracy with low computational cost and fast runtime, making it suitable for research and practical deployment.

### 3.7. Analysis of Failure Cases and Limitations

**Figure 8:**
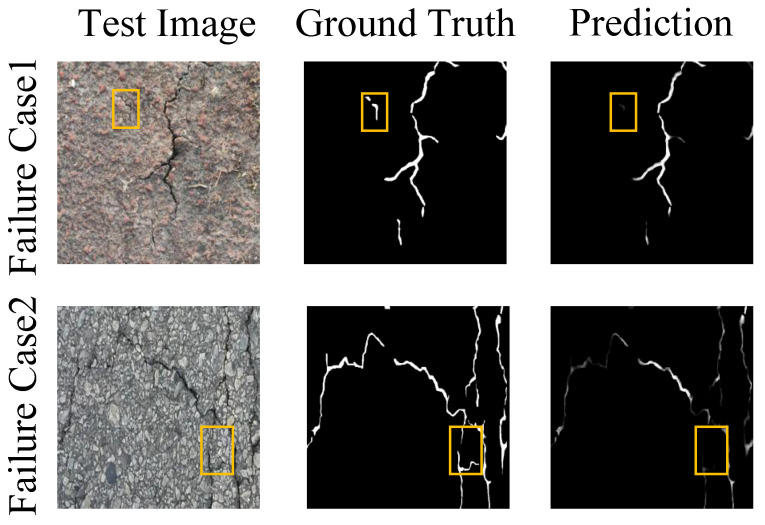
Visualization of CONTI-CrackNet failure cases. Yellow boxes indicate missed detections.

To objectively evaluate the performance of CONTI-CrackNet, we analyze the failure cases observed in our experiments. Figure 8 illustrates two representative scenarios: extreme noisy backgrounds and complex crack intersections. Experiments indicate that in complex scenarios with strong noise and frequent crack intersections, the continuity of fine cracks remains disturbed. Fine cracks may not be fully recovered because complex backgrounds cause confusion between crack pixels and background textures, and intricate crack geometries tend to induce discontinuities at intersections. Although CONTI-CrackNet may exhibit under-segmentation in a few extreme cases, the proposed architecture improves segmentation accuracy under challenging conditions. In future work, we will further enhance the model to increase noise robustness and multi-scale handling, thereby improving the overall performance.

## 4. Discussion and Conclusions

We propose CONTI-CrackNet, which is a lightweight crack-segmentation network that improves pixel-level fine segmentation under low computational cost. Its performance stems from three aspects: (1) a cascaded lightweight backbone that captures crack morphology while reducing resource usage; (2) MD3S, which aggregates multi-directional information to model the global context of thin and irregular cracks; and (3) DBPGL with a PAP mechanism, which employs dual-branch pixel attention with max and average pooling to enhance local details and overall structural perception. On TUT and CRACK500, the model achieves superior or comparable accuracy with 24.22 G GFLOPs and 6.01 M parameters while maintaining high inference speed. Ablation studies show that MD3S strengthens continuity, and DBPGL with PAP improves segmentation by coupling global dependencies with detail enhancement. Limitations remain: validation is conducted on two public datasets and focuses on static images. Future work will expand data diversity, improve small-crack segmentation, investigate crack-depth quantification, and assess pathways toward efficient edge execution.

## Figures and Tables

**Table 1:** Description of the experimental datasets.

Dataset	Resolution	Images	Training	Validation	Test
TUT [42]	640×640	1408	986	141	281
CRACK500 [43]	640×360	3368	2358	337	673

**Table 2:** Sensitivity analysis of α on the TUT dataset.

α	ODS	OIS	F1	P	R	mIoU
0	0.8021	0.8059	0.8279	0.8176	0.8384	0.8368
0.1	0.8049	0.8116	0.8292	0.8185	0.8402	0.8371
0.2	0.8133	0.8165	0.8332	0.8220	0.8447	0.8436
0.3	0.8110	0.8123	0.8313	0.8196	0.8434	0.8420
0.5	0.8002	0.8017	0.8283	0.8201	0.8366	0.8359

**Table 3:** Comparison of segmentation results of different models on the TUT dataset.

Methods	ODS	OIS	F1	P	R	mIoU
SFIAN (2022) [31]	0.7290	0.7513	0.7473	0.7715	0.7247	0.7756
SegNeXt (2022) [11]	0.7312	0.7435	0.7517	0.7812	0.7245	0.7785
Crackmer (2024) [14]	0.7429	0.7501	0.7578	0.7501	0.7656	0.7966
SegFormer (2021) [13]	0.7532	0.7612	0.7670	0.7654	0.7688	0.8078
CT-crackseg (2023) [32]	0.7940	0.7996	0.8199	0.8202	0.8195	0.8301
CSMamba (2024) [22]	0.7879	0.7946	0.8146	0.7947	0.8353	0.8263
PlainMamba (2024) [20]	0.7889	0.7954	0.8154	0.7955	0.8365	0.8316
Ours	0.8133	0.8165	0.8332	0.8220	0.8447	0.8436

**Table 4:** Comparison of segmentation results of different models on the CRACK500 dataset.

Methods	ODS	OIS	F1	P	R	mIoU
SFIAN (2022) [31]	0.6473	0.6941	0.7204	0.6983	0.7441	0.7315
SegNeXt (2022) [11]	0.6488	0.6762	0.7334	0.7134	0.7546	0.7345
Crackmer (2024) [14]	0.6933	0.7097	0.7267	0.6985	0.7572	0.7421
SegFormer (2021) [13]	0.6998	0.7134	0.7245	0.7067	0.7434	0.7456
CT-crackseg (2023) [32]	0.6941	0.7059	0.7322	0.6940	0.7748	0.7591
CSMamba (2024) [22]	0.6931	0.7162	0.7315	0.6858	0.7823	0.7592
PlainMamba (2024) [20]	0.6574	0.6870	0.7422	0.7318	0.7530	0.7579
Ours	0.7104	0.7301	0.7587	0.7333	0.7860	0.7760

**Table 5:** Ablation validating the effectiveness of the proposed modules.

MD3S	ElemAdd	SKNet	DBPGL	PAP	ODS	OIS	F1	P	R	mIoU
✗	✓	✗	✗	✗	0.7845	0.7921	0.8049	0.7945	0.8156	0.8078
✓	✓	✗	✗	✗	0.8008	0.8074	0.8268	0.8161	0.8377	0.8350
✓	✗	✓	✗	✗	0.7944	0.8064	0.8200	0.8123	0.8279	0.8268
✓	✗	✗	✓	✗	0.8094	0.8168	0.8297	0.8142	0.8459	0.8406
✓	✗	✗	✓	✓	0.8133	0.8165	0.8332	0.8220	0.8447	0.8436

**Table 6:** Ablation validating the effectiveness of the proposed MD3S.

Method	ODS	OIS	F1	P	R	mIoU
ParaSna	0.7880	0.7926	0.8063	0.7898	0.8236	0.8041
DiagSna	0.7897	0.7979	0.8077	0.7891	0.8273	0.8059
ParaSna + DiagSna	0.7970	0.8071	0.8127	0.7909	0.8359	0.8260
MD3S	0.8008	0.8074	0.8268	0.8161	0.8377	0.8350

**Table 7:** Ablation validating the effectiveness of the PAM.

Methods	ODS	OIS	F1	P	R	mIoU
CBAM	0.8016	0.8023	0.8250	0.8245	0.8256	0.8277
PAM	0.8133	0.8165	0.8332	0.8220	0.8447	0.8436

**Table 8:** Comparison of CONTI-CrackNet and other methods in Params, GFLOPs, and FPS.

Methods	GFLOPs	Params	FPS
SFIAN (2022) [31]	84.57 G	13.63 M	32
SegNeXt (2022) [11]	31.80 G	27.52 M	22
Crackmer (2024) [14]	14.94 G	5.90 M	33
SegFormer (2021) [13]	30.80 G	28.20 M	21
CT-crackseg (2023) [32]	39.47 G	22.88 M	28
CSMamba (2024) [22]	145.84 G	35.95 M	19
PlainMamba (2024) [20]	73.36 G	16.72 M	33
Ours	24.22 G	6.01 M	42

## Data Availability

Publicly available datasets were analyzed in this study. The TUT dataset can be found at https://github.com/Karl1109/CrackSCF, accessed on 2 July 2025, and the CRACK500 dataset is available at https://github.com/fyangneil/pavement-crack-detection, accessed on 2 July 2025.
